# What is known about gambling in lesbian, gay, bisexual, trans and queer (LGBTQ+) communities? A scoping review

**DOI:** 10.1136/bmjopen-2024-096792

**Published:** 2025-09-14

**Authors:** Laetitia Zeeman, Alexandra Sawyer, Louis Bailey, Nigel Sherriff, Matt Smith

**Affiliations:** 1 School of Education, Sport and Health Sciences, Centre for Transforming Sexuality and Gender, University of Brighton, Brighton, UK; 2 Centre for Transforming Sexuality and Gender, University of Brighton, Brighton, UK

**Keywords:** Health Equity, MENTAL HEALTH, QUALITATIVE RESEARCH, Sexual and Gender Minorities

## Abstract

**Abstract:**

**Background:**

Gambling is of public health importance due to the potential impacts of gambling on individuals and their communities.

**Objectives:**

This review draws on evidence to address: ‘What is known about gambling in lesbian, gay, bisexual, trans and queer+ (LGBTQ+) communities?’ including (i) the prevalence of gambling harm; (ii) the lived experience of gambling harms; (iii) the interventions and service barriers and (iv) the risk and protective factors against gambling harms.

**Eligibility criteria:**

The identified peer-reviewed and grey literature papers were screened against inclusion and exclusion criteria by two reviewers prior to extracting data. Eligibility for inclusion was assessed via the Critical Appraisal Skills Programme (CASP) framework and a Weight of Evidence approach.

**Sources of evidence:**

PubMed, Web of Science, ProQuest, Google Scholar and Cochrane were searched for peer reviewed and grey literature published from June 2000 to June 2023.

**Charting methods:**

Data extraction tables were developed to include the characteristics, methods, sample and key findings for each study.

**Results:**

19 papers were included, which showed mixed prevalence of problems with gambling among lesbian, gay and bisexual populations. There is more consistent evidence that trans and gender diverse people experience higher levels of problems with gambling compared with cisgender (not trans) people. Limited research focused on the lived experience or the wider impact of gambling harm among LGBTQ+communities. Risk factors for gambling harm included minority stress, societal stigma, discrimination and isolation. Protective factors against gambling harm included higher levels of support, positive social interaction and mainstream community connectedness. No studies were identified with gambling interventions specific to LGBTQ+people. General health service barriers included professionals’ use of pathologising language or a lack of cultural competency and education around LGBTQ+issues.

**Conclusion:**

Research on LGBTQ+ gambling harm remains distinctly limited. Further, population-based surveys as well as in-depth qualitative research are needed to develop a comprehensive understanding of gambling in LGBTQ+communities. Research should be undertaken in collaboration with LGBTQ+peers. A better understanding of gambling could inform a whole systems approach with targeted interventions to protect against gambling harm and to promote greater health equity. Open Science Framework registration number (http://osf.io/jf85y/).

**STRENGTHS AND LIMITATIONS OF THIS STUDY**

- The current review included a synthesis of both peer-reviewed and grey literature, with grey literature seen as an important source of research involving minoritised populations in under-researched areas.
- Since this review is framed within a UK context, where there is limited evidence of gambling research with lesbian, gay, bisexual, trans and queer+ (LGBTQ+) people, evidence from comparative legislative, policy and cultural contexts were considered.
- The dearth of research into LGBTQ+ gambling means that data presented in this review, and subsequent interpretations and conclusions, are limited.
- Heterogeneity of included studies in terms of study design, measurement of gambling and measurement of sexual orientation and gender identity makes it difficult to compare across studies and draw robust conclusions about gambling in LGBTQ+populations.
- Data presented in this review were weighted towards the USA, Australia, Canada and Western Europe, which limits generalisability to other settings.

## Introduction

 Gambling is of increasing importance for public health due to the potential impacts of gambling on individuals and their communities. As a recreational activity, gambling can be undertaken without any harmful effects; however, gambling and the associated harm is perceived as a growing public health concern in many parts of the world.^1^ With increasing access to online gambling, the global estimates of revenues raised from online gambling are projected to grow to US$277 billion by 2034.^2^ The prevalence of ‘problem gambling’ varies between international settings.^3^ A recent global review of gambling in 68 countries found that 46.2% (95% CI 41.7 to 50.8) of adults and 17.9% (14.8–21.2) of adolescents had gambled in the past 12 months. These figures equate to an estimated 2.3 billion people worldwide and 159.6 million adolescents worldwide. For adults, 8.7% (6.6–11.3) were found to engage in risk gambling, and 1.41% (1.06–1.84) were engaging in ‘problem gambling’.^4^ Within a UK context, a recent GB survey conducted in England, Scotland and Wales estimated that 61% of adults reported that they had participated in some form of gambling activity over the past 12 months.^5^ The same survey found that overall 3.5% of adults surveyed reported ‘moderate’ levels of problems associated with gambling scoring 3+, and 2.9% of adults surveyed scored 8+ on the Problem Gambling Severity Index (PGSI) – indicating ‘problem gambling’.^6^ Gambling-related harms associated with ‘problem gambling’ may include mental health challenges, as well as relationship problems or financial difficulties for the person and their significant others.^7^ A gambling-related harms framework identified additional areas of harm in relationship disruption, decrements to health and emotional or psychological distress, with reduced performance at work or study, and criminal activity.^8^ As a result, any actions to tackle gambling harms should address resources (work, money, debt and crime), and health (physical and mental) as well as relationships (partners, family, friends and community).^9^

The effects of gambling on different population groups need to be investigated. An estimated 3.3% of the UK population aged 16 years and over identified as lesbian, gay or bisexual (LGB) in 2022, accounting for around 1.8 million people.^10^ People identifying as LGB increased from an estimated 2.1% in 2017 to 3.3% in 2022.^10^ For this expanding population, LGB people along with trans and non-binary people, health inequalities are well documented in systematic reviews of research.^11^,^14^ Analysis of a General Practitioner (GP) patient survey in England found considerable mental health inequalities for LGB people compared with heterosexual peers.^15^ Substance use research indicates that inequalities in harmful alcohol use exist for lesbian, gay, bisexual, trans, queer+ (LGBTQ+) people compared with their heterosexual, cisgender (not trans) peers.^16^

With growing awareness of gambling harms and the range of determinants that underpin gambling, a recent UK-based survey for LGBT and non-binary people in Scotland indicated that among 446 respondents who gambled, 3% experienced ‘problem gambling’, scoring 8+on the PGSI.^17^ No comparisons were available in this study for heterosexual and cisgendered (not trans) peers. Given the growth of the online gambling industry, larger UK-based surveys are needed to determine the prevalence of gambling and gambling harms for different LGBTQ+ groups, including gender diverse youth. This information is important to inform development of culturally sensitive and inclusive prevention, intervention, and outreach programmes.^18^

However, understanding of gambling in LGBTQ+communities is limited. There is some evidence to suggest that LGBTQ+people may experience slightly higher levels of problems with their gambling compared with heterosexual, cisgender populations. The GambleAware Annual Treatment & Support survey for England, Scotland and Wales found that compared with the general population, more LGB+ respondents reported experiencing problems with their gambling as defined by a PGSI score of 1+ (29% vs 21%).^19^ Several international gambling reviews related to sexual orientation and gender identity exist, including a systematic review^20^ and scoping review.^21^ However, these reviews of gambling in sexual orientation and gender identity minority people found little high-quality research in the field. The first review^21^ was limited to six studies only, and the second review^20^ included studies comprising cross-sectional methodologies only. Both reviews included studies using a range of data, collected either via surveys or interviews and/or secondary data analysis through archives.^20 21^ These reviews identified the need for interventions research with experimental methods and longitudinal follow-up. Both reviews argued for clearer conceptualisation of problem gambling and standardised measurement of gambling-associated variables in the LGBTQ+ groups. Review findings showed conflicting results on the prevalence of gambling in LGBTQ populations^21^ and/or sexual and gender minority (SGM) groups.^20^ Neither review included grey literature, which can be of value to identify current knowledge in under-researched areas and communities, and to complement a review of peer-reviewed literature.^22^ Grey literature may present a rich source of up-to-date information or relevant expert knowledge and is often produced by and for communities themselves, which is beneficial when considering minoritised populations. Due to the limited research and understanding of gambling in LGBTQ+ communities, this scoping review examined both peer-reviewed and grey literature to assess the prevalence of gambling and the related impacts on LGBTQ+ people. Gambling research in comparative legislative, policy and cultural contexts were considered, where there was a lack of evidence in the UK.

### Aims of the review

Our review addressed the following question: *What is known about gambling in the LGBTQ+communities?* The overall aim of this review is to summarise available evidence regarding gambling in LGBTQ+people including:

- the prevalence of gambling among LGBTQ+communities;
- the risk factors for and the types of gambling harm (eg, financial harm, negative impact on relationships and work);
- protective factors that mitigate harm in these communities;
- the lived experience of LGBTQ+people and
- the barriers LGBTQ+people may experience in accessing services and healthcare provision for gambling harm.

## Methods

The review systematically identified all available research on gambling and associated harms in LGBTQ+communities. Due to the heterogeneity of the study focus area involving a range of groups (LGBTQ+) and the available evidence, a full systematic review or meta-analysis was not conducted. Instead, a scoping review methodology^23^ was chosen to capture the breadth of information on gambling. The scoping study was informed by the Preferred Reporting Items for Systematic Reviews and Meta-Analyses guidelines for Scoping Reviews (PRISMA-ScR).^24^ A concise version of the protocol was registered via the Open Science Framework (http://osf.io/jf85y/).

### Inclusion and exclusion criteria

A PICO approach was used to formulate the search question and guide inclusion/exclusion criteria: Population (LGBTQ+communities), Intervention or Phenomenon of Interest (gambling/gambling harm), Comparison or Context (UK and international, with comparable policy and cultural contexts), and Outcome. Inclusion and exclusion criteria were used for both peer-reviewed research and grey literature. Inclusion criteria were papers written in English, published between June 2000 up to June 2023, with a focus on gambling/harms and LGBTQ+ communities (including men who have sex with men; MSM). Primary qualitative, quantitative or mixed methods studies were eligible. Studies were excluded where sexual orientation and gender identity were not clearly defined, where there were no meaningful outcomes or where papers were solely opinions or editorials.

### Literature search

Searches of peer-reviewed literature followed by grey literature searches were conducted between June and October 2023. The searches were undertaken by one author (LB) and verified by another author (LZ) with the support of a university librarian. Titles and abstracts were screened and verified by two authors. Search terms and appropriate synonyms (MeSH terms) for both searches (ie, peer review and grey) included: (“LGB” OR “lesbian” OR “gay” OR “bisexual” OR “trans” OR “transgender” OR “transsexual” OR “queer” OR “non-binary” OR “MSM” OR “intersex” OR “gender identity” OR “sexual orientation” OR “gender minorit*” OR “sexual minorit*”) AND (“gambl*” OR “betting” OR “lotter*” OR “lotto*” OR “casino*” OR “loot box*"). The literature search strategy can be found in online supplemental appendix 1.

#### Peer-reviewed literature

Database searches of international data were made in PubMed, Web of Science, ProQuest and Cochrane. In addition, searches were undertaken in Google Scholar (first ten pages). Reference trails of systematic reviews and other relevant studies were followed to ensure the most relevant papers were found. A total of 16 journal papers were identified for inclusion.

**Figure 1:**
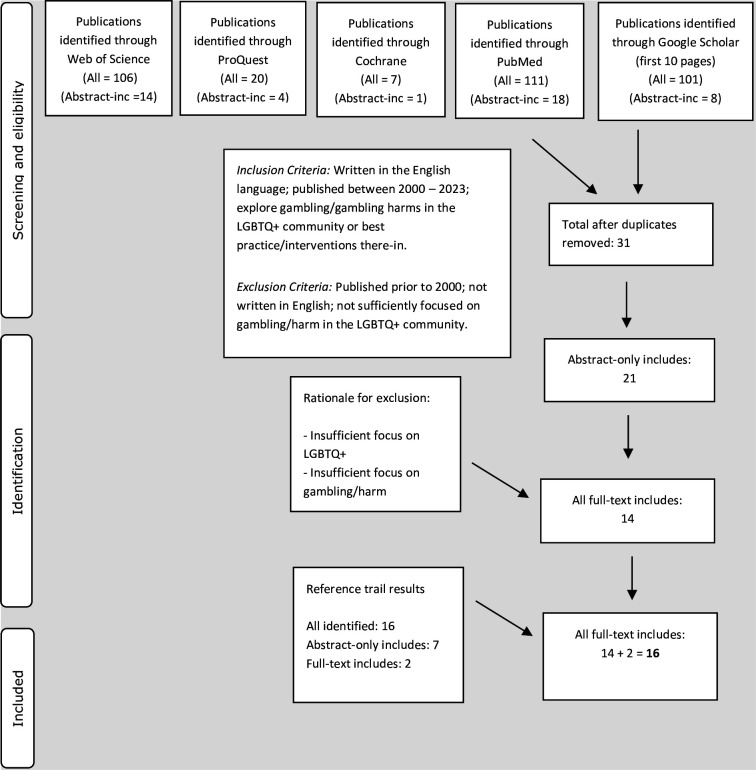
PRISMA-ScR flow diagram of peer-reviewed data. LGBTQ+, lesbian, gay, bisexual, trans and queer+; PRISMA-Scr, Preferred Reporting Items for Systematic Reviews and Meta-Analyses guidelines for Scoping Reviews

Figure 1 displays the PRISMA-ScR flow diagrams of the screening results for peer-review data.

#### Grey literature

In addition to database searches of peer-review articles, searches were undertaken to identify grey literature. This included government reports, third sector research, conference papers, theses and unpublished works. The grey literature synthesis was confined to the UK and other countries within the Organisation for Economic Corporation and Development (OECD) to ensure a comparable legal and policy framework for interpretation. Literature was identified through (i) Google Advanced Search (first 10 pages); (ii) Google searches of relevant LGBTQ+/gambling charity websites; (iii) reference trail searches and (iv) expert recommendations. Following application of the exclusion criteria, three grey literature papers were included. The number of papers included in this review for both peer-reviewed (n=16) combined with grey literature (n=3) was 19 in total.

### Data extraction

Once studies were identified via the searches, database management software (EndNote) was used to allow storage of the primary research citations, to keep track of papers, to identify included and excluded studies and to detect duplicates. Descriptive analysis was conducted via a master table in Word containing key information from each of the selected studies including health topic, time range of year published, geographical scope, the LGBTQ+sub-population, methods employed, scientific journal or grey literature etc. While there is ongoing deliberation in the literature regarding the need for quality assessment of included studies during the scoping review process,^25^,^27^ critical appraisal of the literature is important to enable identification of strengths and limitations of the evidence base. Accordingly, since two types of evidence were included (peer review and grey literature), two methods of critical appraisal were applied.

### Quality assessment of peer-reviewed literature

Quality of the peer-reviewed papers was assessed between the first three authors using an adapted version of a Critical Appraisal Skills Programme (CASP) questionnaire. A visual representation of the appraisals is presented in online supplemental appendix 2.

### Quality assessment of grey literature

A weight of evidence (WoE) approach was used, drawing on Gough^28^ to promote methodological and thematic fairness of grey literature with an applied outcome. This is key given the nature of the review on a 'below the radar’ issue with a marginalised population group and a commitment to improving policy or practice. Papers were assessed using these questions: (1) Do the study findings answer the study questions? (2) Is the research design and analysis appropriate to the aims and objectives of the research? and (3) Is the study relevant to this review? Does the study assist us in addressing the review questions? The answers were combined to provide a WoE rating – low, medium, high, with only studies ranking high^29 30^ or medium^31^ included in the review. Leven *et al*^17^ was excluded based on the weight of evidence due to the health needs assessment focus for LGB and TNB (trans and non-binary) people while containing only a small section on gambling prevalence. As part of the appraisal process, peer-reviewed and grey literature was subject to additional scrutiny around language and the framing of LGBTQ+ identities, lives and experiences, as well gambling and the associated harms. Papers that were found to use stigmatising language were subject to additional critique to ensure these framings are not replicated in this review.

The included papers were combined to form a thematic construction based on the review questions. Themes formed an analytic framework to provide an overview of the breadth of the literature. The thematic analysis that follows is presented as a narrative synthesis.

## Results

Nineteen papers were included from peer reviewed (n=16) and grey literature (n=3). Key characteristics and summaries of these studies are presented in online supplemental appendix 3.

While there is limited research on gambling harms in LGBTQ+populations, interest in this topic has grown in recent years. Just over half of the final included papers (10 out of 19) were published between 2021 to 2023. Around half of the studies included in the review drew on data from the USA (n=9), followed by Australia (n=3) and Sweden (n=3). Other countries included Canada, Switzerland, England, Denmark, Spain, Italy and Poland (one study focused on several countries). All papers drew on cross-sectional surveys (n=19). In addition, only two articles (grey literature) drew on survey data combined with in-depth interviews.^29 31^ Where validated measures of harmful gambling were utilised, this included use of the following: Gambling Disorder Measure (GAM-DS); PGSI; NODS-CLiP 'problem gambling'; Gambling Activities Questionnaire (GAQ); DSM-4 and DSM-5 (Gambling Disorder Symptomatology); Yale-Brown Obsessive Compulsive scale for pathological gambling (PG-YBOCS); Canadian Problem Gambling Index (CPGI); Brief Adolescent Gambling Screen and the Short Gambling Harms Screen.

The topics covered in the papers include prevalence of gambling, gambling type, gambling behaviour, gambling severity and factors associated with gambling such as harms. A range of different gambling activities were explored, including lottery or raffle tickets, card or dice games, instant online games, electronic gambling machines either in person or online, casino table games (online or in person), sports or animal betting, bingo, stocks and other forms of speculative financial market activities. Only one study examined intersecting markers by considering gambling behaviour of sexual orientation and gender identity minority people alongside their migrant or refugee status.^32^

Except for studies by Bush-Evans^31^ and Birch *et al*^33^ which focused *only* on the experiences of the LGBTQ+ population, most of the remaining studies compared gambling prevalence in the LGBTQ+ population to a cisgender, heterosexual sample. Where studies focused only on sub-groups within the LGBTQ+ population, this included findings on prevalence among sexual minority men (n=3) and the trans and non-binary population (n=2).

At the time of writing, findings from two mixed-methods research studies were anticipated – by Brodeur *et al* (Canada) and by Bush-Evans (UK). A policy briefing summarising some of the key findings from Bush-Evans^31^ were included in the synthesis of grey literature presented here, while only a study protocol from Brodeur *et al* was available at the time of conducting the review.^34^

The following themes were identified from across the selected studies: (1) the prevalence of gambling among LGBTQ+communities, (2) the types of harm experienced, (3) the risk factors for gambling harm, (4) the protective factors against gambling harm, (5) LGBTQ+lived experience of gambling and finally (6) service barriers.

### The prevalence of gambling among LGBTQ+ communities

16 papers explored gambling prevalence for LGBTQ+people.^1830 32^,^40 40^ The results of the review yielded mixed evidence regarding prevalence of gambling and the associated harms among SGM populations. However, as not all studies included comparator groups in their data, only studies with relevant comparisons between LGBTQ+groups and heterosexual or cisgender groups were included in the summary. The key findings for prevalence in sexual minority groups are summarised.

#### Sexual minority groups

Broman *et al* found that sexual minority status was a statistically significant predictor of gambling harms (as measured by the NODS-CLiP screening instrument) among sexual minority women (OR 1.3; p=0.001), but not among sexual minority men (OR 0.75; p=0.164), compared with the general population (n=10 983).^35^ Results were mixed when comparing gambling severity according to the PGSI between sexual minority and heterosexual groups. For example, Bush *et al* found that compared with cisgender heterosexual participants, lesbian, gay, bisexual, trans, intersex, queer+ (LGBTIQ+) participants were significantly more likely to be classified in the ‘non-problem’ gambling category (28.5% vs 13.2%) and significantly less likely to be classified in the ‘problem gambling’ category (27.9% vs 39.4%); n=385.^29^ In addition, Bush *et al* reported a slightly higher (non-significant) level of ‘problem gambling severity’ (8+on the PGSI) in heterosexual men (n=207) when compared with sexual minority men (n=101) 39.7% compared with 34%.^36^ Although heterosexual men reported higher levels of ‘problem gambling severity’ compared with sexual minority men, the gambling-related harms did not differ between groups.

However, in the findings of Richard *et al*^30 43^ with a sample of US college student athletes, gay and bisexual men had ‘problem gambling’ symptomatology that was 3.42 times higher than heterosexual men (p<0.01), while gay/lesbian women (n=274) and bisexual women (n=303) had ‘problem gambling’ symptomatology that was 2.57 times higher than heterosexual women (n=8215; p<0.01).^43^ Here, ‘problem gambling’ was measured by the DSM-5 diagnostic criteria for the ‘gambling disorder’ symptomatology.

A Canadian health survey found that sexual minority men (n=3 08 600) were revealed to have higher rates of ‘problem gambling’ scoring >3 on the Canadian Problem Gambling Index (CPGI) compared with heterosexual men (n=9,353,000) via an adjusted OR of 3.0 vs 1.0; (95% CI; p<0.05).^30^ The percentage of people who gambled in the past year at moderate-to-severe risk of ‘problem gambling’, was higher in the sexual minority sample group compared with the heterosexual group (2.1% vs 1.0%).^30^ Here, ‘moderate risk’ was indicated by a CPGI score of between 3 and 7, while ‘severe risk’ scores were >8.

Young American LGB adults (n=51) aged 18–29 reported significantly more symptoms of 'problem gambling’ over the preceding week (7.88% vs 4.72%; p<0.01) compared with non-LGB young adults (n=483) as measured via the PG-YBOCS: Yale-Brown Obsessive–Compulsive Scale Modified for Pathological Gambling.^37^ Tables1^3^ present summaries around prevalence of gambling.

**Table 1:** Provides a summary of findings around prevalence of gambling and sexual orientation from across the peer-review literature

Research peer reviewed	Non-LGB	LGB	Bi-sexual	Hetero sexual women	Sexual minority women	Hetero- sexual men	Sexual minority men	Outcome or measure
Broman & Hakansson^35^ (2018)	10% (n=543)	11% (n=77) (p=0.85, *ns*)						NODS-CLiP problem gambling
Broman *et al*^54^ (2022)				13,5% (n=3948)	20.4% (n=735) p-value 0.001	23.6% (n=874)	26.2% (n=84) 23.6% (n=874) No statistical significance OR 0.75; p-value 0.164)	NODS-CLiP problem gambling
Bush *et al*^36^ (2021)						39.7% (n=207)	34% (n=101)	PGSI score for problem gambling 8+, but no statistically significant effect for gambling harms
Grant & Chamberlain^37^ (2023)	4.72% (n=483)	7.88% (n=51)						Yale Brown obsessive-compulsive scale for pathological gambling
Hershberger and Bogaert,^39^(2005)				<1% (n=3250) p<0.05	<1% (n=785)	<1% (n=1051)	<1% (n=8115)	Two questions to assess current frequency of gambling *figures presented here relate to high levels of gambling
Richard *et al*^43^ (2019)			women 0.44% (n=303) men 2.07% (n=65)	0.26% (n=8215)	0.22% (n=274)	0.81% (n=10 305)	0.63% (n=137)	Gambling activities questionnaire (GAQ)+DSM-5 gambling disorder
Wicki *et al*^44^ (2021)		3% (n=335)	2.3% (n=56)			1.1% (n=4722)	1.7% (n=125)	Mild gambling-use disorder based on DSM-5 criteria

LGB, lesbian, gay or bisexual; PGSI, problem gambling severity index .

**Table 2:** Provides a summary of findings around prevalence of gambling and sexual orientation from across the grey literature

Research grey literature	Hetero-sexual women	Hetero-sexual men	Sexual minority men	Sexual minority women	Cisgender hetero-sexual	LGBTQI+	Outcome or measure
Bush *et al*^29^ (2020)					39.4% (n=207)	27.9% (n=110)	PGSI score for problem gambling 8+
Rotermann & Gilmour^30^ (2022)	1.0%	1.0% (n=9,353,000)	3.0% (n=3 08 600)	0.8%			Canadian problem gambling index (CPGI)>=3

LGBTQI+, Lesbian, Gay, Bisexual, Transgender, Queer or Questioning and Intersex; PGSI, problem gambling severity index.

**Table 3:** Summary of findings around prevalence of gambling and gender identity from across the peer-review literature

Research peer reviewed	Cisgender youth	Trans/ gender diverse (TGD) youth	Migrant sexual/ gender minority	Refugee sexual/ gender minority	Refugee heterosexual	White sexual/ gender minority	White hetero sexual	Outcome or measure
Mattelin *et al*^32^ (2022)			9.6% (n=285)	5.2% (n=253)	5.9% (n=4194)	6.7% (n=4300)	3% (n=1 43 694)	PGSI>1+
Noel *et al*^41 42^ (2022)	1.32% (n=546)	3.61%						Rhode Island Young Adult Survey (RIYAS)
Rider *et al*^18^ (2019)	1.8% (n=23 081)	5.7% (n=571)						Brief Adolescent Gambling Screen (BAGS) 4+

PGSI, problem gambling severity index.

#### Gender minorities

A large-scale population study of adolescents in America (n=80 929) found that trans and gender diverse youth had higher rates of ‘problem gambling’ compared with their cisgender peers (5.7% vs 1.8%; p<0.001; d=0.08)^18^ with scores of 4+on the Brief Adolescent Gambling Screen (BAGS). According to birth-assigned sex, trans/gender diverse (TGD) youth assigned male at birth were particularly at risk of ‘problem gambling’. For example, 8.9% of TGD youth assigned male at birth were experiencing considerable levels of gambling harms compared with rates of only 1.0–2.1% for both cisgender male and female youth. Higher rates of TGD youth assigned female at birth (with a BAGS score of 2+) required further assessment for gambling harms compared with cisgender youth assigned female (7.6% vs 2.2%;p<0.001, d=0.14).^18^

Elsewhere, for young American people aged 18–25 (n=546), the prevalence of ‘problem gambling’ symptoms was 11.4% in the overall population of young people, and the only statistically significant variance was found among trans young people, where the prevalence was 3.6 times higher as compared with other gender sub-groups: 95%CI=1.32, 9.86 (n=546).^41^ Prevalence was measured according to the Rhode Island Young Adult Survey (RIYAS).

Ethnic minority trans refugees in Sweden had the highest odds for risk gambling measured via the PGSI with scores of >1 compared with other sub-groups (White: OR1.48, 0.66–3.31, migrant: OR1.42, 0.72–2.82 and refugee OR8.62, 1.94–38.40).^32^ Figures are presented in table 3.

### Types of harm experienced across LGBTQ+ communities

Few studies examined the impact of gambling harms across LGBTQ+communities such as getting into debt, negative impact on relationships or work, decrements to health, and cultural harm. Bush *et al*^29^ measured gambling-related harms via the Short Gambling Harms Screen,^45^ which captures financial, emotional/psychological and relationship impact due to gambling, and reported that the LGBTQ+ population appeared to experience fewer gambling harm-related impacts when compared with a cisgender, heterosexual sample. Where comparisons were made around bisexuality and gambling harm impact in relation to academic performance, drawing on bisexual college students, the evidence suggests that bisexual women may experience fewer impediments to academic performance due to gambling compared with bisexual men.^46^

### Risk factors for LGBTQ+ gambling

Several studies examined risk factors for gambling harms within the LGBTQ+population.^29 32 33 36 37 47^ However, most studies were cross-sectional and therefore do not demonstrate causality.

There is emerging evidence to suggest that perceived sexual and gender identity-related stigma may play a role in terms of gambling-related harms as experienced by sexual minority men.^36^ Sexual minority men who experience greater perceived stigma based on sexual orientation and gender identity gambled at more ‘problematic’ levels and experienced increased gambling-related harms, p<0.05 (*ibid*). As such, minority stress may play a role in gambling harms experienced by sexual minority men.

A study on TGD youth^18^ described how young people experience minority stress linked to their gender identity, where their gender assigned at birth and their preferred gender expression do not align. Puberty is seen as a difficult developmental period for trans and gender diverse young people due to physical and hormonal bodily changes where there may be some level of dissonance between their body and their gender identity. When their gender identity and the related gender expression are not affirmed, some young people may use risk-taking behaviour and/or gambling to escape the emotional pain, or to gain acceptance from others who engage in similar activities. For some, this may lead to gambling harm.^18^

Higher levels of drug and alcohol use were linked to gambling harms in LGBTQ+people^33^ and in SGM migrants and refugees.^32^ Similar findings were reported from interviews with LGBTQ+people engaged in gambling, which revealed shared pathways to gambling through themes of substance use, mental health challenges, and other life stressors (similar to cisgender heterosexual groups). Where differences were observed in relation to LGBTQ+status, this was focused in the area of gambling beliefs. The further Australian survey with multiple hierarchical regression analyses for the cisgender heterosexual participants and for the LGBTQ+group showed that perceived inability to stop gambling emerged as a more pronounced risk factor for 'problem gambling' severity among LGBTQ+respondents compared with their cisgender, heterosexual peers.^29^ In addition, anticipating gambling success was more pronounced among LGBTQ+ participants, predicting both higher 'problem gambling' severity and related harms.^29^ Elsewhere, Grant and Chamberlain found that LGB young adults were significantly more likely to gamble for longer periods of time (p<0.01), and for more money (p<0.01), compared with non-LGB young adults.^37^ The authors proposed in this sample, LGB people experienced a constellation of behaviours such as nicotine use, substance use and anger which may put them at risk of gambling.^37^

There were mixed results regarding the relationship between psychological distress and gambling harms within LGBTQ+communities, with Bush *et al* finding a significant association with 'problem gambling' severity and associated harms among sexual minority men where more severe gambling resulted in greater harm.^36^ However, Birch *et al* found that mental health variables, such as depression and anxiety for adult LGBTQ+people were not related to gambling harms across both SGM groups.^33^ Another study of LGB youth who were at risk of gambling harms found associated risk factors such as increased suicidality and obsessive–compulsive disorder.^37^ Elsewhere, adult trans respondents who had experienced suicidal ideation were no more likely than other trans individuals to report difficulties with gambling.^47^ In addition, no differences emerged in relation to the gambling habits of trans respondents who had attempted suicide and those who had not (*ibid*).

### Protective factors against gambling harms among LGBTQ+ communities

Findings from included research indicate that the social networks of the LGBTQ+population play a significant part in mitigating against gambling harms. The role of social support emerged as a significant protective factor in the LGBTQ+population across two different papers based on the same study.^29 36^ Greater social support was significantly associated with lower levels of 'problem gambling' severity as measured via the PGSI, and lower levels of gambling-related harms as indicated via Short Gambling Harm Screen scores.^29^ The results of bivariate analyses found that higher levels of emotional support, positive social interaction and mainstream community connectedness predicted lower levels of gambling harms for sexual minority men, but not for heterosexual cisgender men.^36^

### LGBTQ+ gambling lived experience

Qualitative data on the lived experience of gambling in LGBTQ+groups is sparse with only two of the included studies (both grey literature articles) using a qualitative approach to understanding gambling in LGBTQ+ communities. This is particularly surprising given how important qualitative research is for understanding the realities and specific needs of minority communities. The included studies by Bush *et al* in Australia^29^ and Bush-Evans in the UK,^31^ both drew on survey and interview data. There was evidence from both studies showing that for some LGBTQ+ participants, gambling acted as a form of escapism, when associated with the stress and fear of coming out.

If you’re in the closet and you just want to escape from reality, it [gambling] is a route to escape.^31^

Maybe it’s the stress of actually coming out and maybe at that time I couldn’t cope with it very well. I just decided to, at the time, just do that gambling thing instead of actually focussing on what I actually needed to focus on.^29^

These quotations demonstrate the connection of gambling, identity and subjective reality, with gambling used as a form of escapism and as a means of dealing with the fallout from adverse life events. Here, gambling acts as a vehicle to ensure adverse life stressors are manageable; however, given gambling is associated with myriad harms, its use as an escape is not without risk. Vulnerability to gambling harms may be seen to follow on from certain risk factors that preceded. These risk factors reflected in the literature included minority stress, societal stigma and/or discrimination, isolation and victimisation that was framed in some instances as hate crime:

If someone’s been a victim of hate crime that’s something that can potentially get them into the habit.^31^

In Bush *et al* the theme of secrecy came up both in relation to participants’ sexual and gender identity and regarding their gambling behaviour, as shown by the following quotation:

One of the things that always get reinforced is the fact that they are very good at hiding and so, and that sort of is in conjunction with gambling which is also something that people hide. So it’s become like a double risk factor because they are so expert in hiding their sexuality. So it’s not so hard for them to hide their gambling as well.^29^

The above indicates that LGBTQ+people may experience multiple stigma and associated shame both on account of their sexual orientation or gender identity and based on their gambling behaviour. As such, LGBTQ+people may be compelled to keep their gambling hidden and to avoid sources of potential help from friends or formalised services. As many LGBTQ+people did not feel safe in traditional gambling settings or when attending in-person venues, online gambling was viewed as more inclusive and accessible due to the virtual environment offering some form of anonymity.^31^

### Service barriers and LGBTQ+ people

Information on LGBTQ+ gambling service user experiences and the associated barriers that prevented access to care was limited. Bush *et al*, in the second part of their study with LGBTQ+ people, explored via in-depth interviews the experiences of participants’ when accessing gambling-related support services.^29^ The study reported that none of the respondents with ‘problem gambling’ had accessed gambling support services (n=13). Instead, commentary centred on barriers experienced within mental health and social services and, in particular, a lack of awareness, education and cultural competency concerning LGBTQ+ issues among health and social care professionals.^29^ Interviews with stakeholders (ie, LGBTQ+ health workers, gambling support workers) suggested that LGBTQ+ people’s experience of stigma and discrimination due to their sexual orientation or gender identity might act as a *“double barrier”* to help seeking.^29^

Other barriers LGBTQ+ people encountered in these gambling services included where professionals used pathologising language based on the gender identity and/or sexual orientation of service users and asked inappropriate questions.^29^ Related to the use of language, Mattelin *et al*, found barriers to accessing care for SGM migrants and refugees included an inability to speak the language (Swedish), and experiences of stigma due to their minority status, or SGM participants were faced with heteronormative attitudes.^32^ SGM migrants and refugees had limited awareness of services available and how to access these services. Hence, there is a need to increase care access to services for SGM people, as well as developing interventions specifically tailored to meet their needs.^32^

## Discussion

### Key review findings are as follows in terms of the prevalence and gambling harms

This review found mixed evidence about the prevalence of gambling and gambling harms among sexual minority populations. There was some evidence of higher burdens of gambling harms among sexual minority individuals, particularly in relation to men and LGBTQ+youth. However, other studies showed that sexual minority individuals reported lower prevalence, and some studies reported no association between sexual orientation and gambling. There is, however, more consistent evidence that TGD people might experience higher levels of gambling harms compared to cisgender people. There is limited research focussing on the lived experience of gambling harm among LGBTQ+communities. In addition, few studies have examined the wider impact of gambling (e.g., financial harm, impact on relationships and work) among LGBTQ+people.^36^

### Risk, protective factors and gambling-related support

The literature highlighted risk factors including minority stress, societal stigma and/or discrimination, isolation and victimisation. Emerging evidence suggests that perceived stigma may play a role both in terms of the severity of gambling harms experienced and related impact among sexual minority men.^36^ Thus, where gender identity and sexual orientation intersect, the perceived stigma associated with their minority status may contribute to the gambling harms experienced by men. There is some evidence to suggest that general anxiety around everyday disclosures of gender identity or sexual orientation, and anticipated stigma, may be a risk factor for gambling harms, where gambling offers a form of escapism. Evidence linked higher levels of drug and alcohol use to gambling harms.^32 33^ In terms of protective factors, higher levels of support, positive social interaction and mainstream community connectedness predicted lower levels of gambling harm for sexual minority men, but not for heterosexual cisgender men.^36^ Accordingly, social support emerged as a protective factor unique to the LGBTQ+ population.^29 36^ No studies looked at services or interventions specific to LGBTQ+ people experiencing gambling harms, while the limited research available on services focused on accessing mental health and social care services in general.^29^ General health service barriers included professionals’ heteronormative attitudes that became apparent in the use of pathologising language, and/or a lack of cultural competency and education for health professionals on LGBTQ+ issues.^29 32^

When interpreting the findings against broader literature, minority stress theory^48^ suggests populations such as LGBT Q+people experience stress where their non-normative gender identity or sexual orientation is not affirmed. This may in turn, contribute to behaviour associated with risks and harms, including gambling. Here, gambling could offer avenues to gain acceptance while escaping emotional pain.^49 50^ Reviews of research have shown that inequalities in health outcomes for LGBTQ+ people beyond just gambling exist due to the consequences of a range of factors including minority stress associated with sexual orientation and gender identity, cultural and social norms that preference and prioritise heterosexuality and binary gender and LGBTQ+-based victimisation, discrimination and stigma.^51^ A large UK national survey found at least two in five (40 %) LGBTQ+ people had experienced verbal harassment or physical violence in the 12 months preceding the survey.^52^ Thus, efforts to address inequalities in health outcomes (including gambling) must tackle the social determinants of inequalities at an individual level, as well as addressing broader structural factors as part of a whole systems approach. As little is known about gambling in LGBTQ+ people, and these research findings raise several questions. Do LGBTQ+ people experience a greater burden of gambling harm due to their minority status and what are the drivers of gambling harms for these communities? Increased understanding of gambling in LGBTQ+ communities can inform the development of culturally sensitive and inclusive prevention, intervention and outreach programmes.^18^ These programmes should emphasise understanding of the cultural and social contexts of LGBTQ+ communities to tailor interventions effectively, as well as focusing on reducing minority stress related to the impacts of stigma, homophobia, transphobia and other forms of discrimination.^51^ Culturally sensitive programmes should be inclusive and reflect the diversity of LGBTQ+ communities to ensure programme content is appropriate and specific to the intended participants. By using an intersectional approach, overlapping identities and experiences of LGBTQ+people such as sexual orientation, gender identity, age and ethnicity could ensure that interventions are comprehensive and address the specific needs of subgroups within these communities. Culturally sensitive interventions would aim to create supportive environments by providing tailored resources to help LGBTQ+ people and service providers tackle gambling-related harms.^53^

### Strength and limitations

Sexual orientation and gender identity are complex multifaceted concepts that have posed challenges for public health researchers engaged in investigating the health inequalities of LGBTQ+people. When examining gambling in LGBTQ+communities, ideal prevalence evidence for gambling harm in gender and sexual minority communities would be from an adult national sample selected randomly that measured sexual orientation and gender identity and presented results disaggregated by sex, gender and sexual orientation compared with a heterosexual and cisgender majority. There would be calculations presented as to whether any difference in prevalence is statistically significant between various groups. At the time of writing the paper, this kind of evidence did not exist in the UK for the research included in the review. As a further consideration in studies using quantitative methods, subdivision of a sample by sexual orientation and gender identity may yield such small group sizes that parametric tests would not have adequate power to achieve statistically significant results. As literature reporting on LGBTQ+gambling prevalence in the UK is sparse, the review drew on prevalence data for research undertaken in the UK where available, and within comparable legislative and cultural frameworks.

The current review included the synthesis of both peer-reviewed and grey literature. Previous reviews appear to have excluded grey literature^16 17^; however, it can be an important source of research involving minoritised populations as well as under-researched areas. Although only three studies were included from the grey literature search, these nevertheless provided useful insight into the prevalence of gambling in LGBTQ+people,^30^ experiences of gambling, as well as risk and protective factors presented in mixed-methods research.^29 31^ Indeed, the only qualitative research included in this review was identified in the grey literature search. The overall lack of research into LGBTQ+ gambling harms means that data presented in this review, and subsequent interpretations and conclusions, are necessarily limited. Moreover, the heterogeneity of included studies in terms of study design, measurement of gambling, and measurement of sexual orientation and gender identity makes it difficult to compare across studies and draw robust conclusions about gambling in LGBTQ+ populations. Studies presented in this review were weighted towards the USA, Australia, Canada and Western Europe, which limits generalisability to other settings.

### Implications

The overriding implication of this review is that more robust, respectful and culturally competent research is needed to understand gambling and gambling harms in LGBTQ+populations in the UK, and arguably more broadly. There is a need for large-scale, population-based surveys to estimate the prevalence of gambling and gambling harms in LGBTQ+populations. Qualitative, ethnographic and/or longitudinal research is needed to establish a picture of gambling harms across the life course, and to identify the risk and causal factors for gambling harms in LGBTQ+people, as well as the protective factors against gambling harm. This review also highlights the need for standardised and robust monitoring of gender identity and sexual orientation and linked data collection, which separates gender identity from sex assigned at birth, and which considers the unique needs and perspectives of the various minority groups within the LGBTQ+umbrella. Finally, this review did not identify any research which explored help-seeking for gambling in LGBTQ+ populations, or any interventions targeted at LGBTQ+ people experiencing gambling harms, though it did identify some of the barriers experienced by these communities when accessing services. Therefore, there is a growing need for evidence-based interventions to address gambling harms in LGBTQ+people.

## Conclusion

This scoping review summarised the available literature on gambling in LGBTQ+ populations. However, the sparse evidence and inconsistent findings limits what can be concluded about gambling in LGBTQ+ populations. The mixed findings around prevalence highlight the need for large-scale, population-based surveys using validated measures of gambling. This would be particularly valuable to identify risk and causal factors, which are essential to the development of prevention work and targeted interventions for LGBTQ+ populations. Consistent data collection around gender identity and sexual orientation is crucial to produce meaningful and robust new research. In addition, there is a need for qualitative research around lived experiences of LGBTQ+ people, their gambling behaviour and the linked harms. Future research should prioritise the LGBTQ+population, including the sub-groups within, who are minoritised and who may experience multi-layered levels of vulnerability and risk. Any research should be undertaken with greater community involvement and in collaboration with LGBTQ+ peers. Improved understanding of gambling could inform a whole systems approach with health promotion initiatives and the development of targeted interventions to protect against gambling harm in LGBTQ+communities and to ultimately achieve greater health equity.

## Supplementary material

10.1136/bmjopen-2024-096792 online supplemental appendix 1

10.1136/bmjopen-2024-096792 online supplemental appendix 2

10.1136/bmjopen-2024-096792 online supplemental appendix 3

10.1136/bmjopen-2024-096792 online supplemental file 1

## Data Availability

All data relevant to the study are included in the article or uploaded as supplementary information.
