# Supplementary material for: What is known about gambling in lesbian, gay, bisexual, trans and queer (LGBTQ+) communities? A scoping review
Source: BMJ Open. 2025 Sep 14;15(9):e096792. doi: 10.1136/bmjopen-2024-096792 (PMC12434734; doi:10.1136/bmjopen-2024-096792)
Supplement: online supplemental appendix 1 [file bmjopen-15-9-s001.docx]

**Appendix 1: Literature search strategy**

The search strategy defined where to search, which terms to use, which sources were searched, time span, and language(s). Example sources included electronic databases, reference lists, hand searching of organisations, and websites. Although breadth and practicalities of the search were important, we proposed viable parameters upfront, with clear inclusion and exclusion criteria (Arksey and O’Malley 2005). The search was conducted with the support of a university librarian. Targeted searching ensured each search was specific with a smaller number of hits but a higher likelihood of relevant papers. However, sensitivity of the search (broad search scope with a large number of hits) was balanced with specificity (narrow targeted search).

Database searches were undertaken for published literature.

**Search 1** looked for UK evidence published in **peer reviewed** journals in English between June 2000 – June 2023 on:

- the prevalence of gambling amongst LGBTQ+ communities;
- the risk factors for and the types of gambling harm (e.g. financial harm, negative impact on relationships and work);
- protective factors that mitigate harm in these communities;
- the lived experience of LGBTQ+ people; and,
- the barriers LGBTQ+ people may experience in accessing services and healthcare provision for gambling harm.

Inclusion criteria based on the PICOS framework are, 1) population - gender and sexual minority / LGBTQ+ people, 2) intervention – gambling / gambling harm, 3) comparator - compared to heterosexual and cisgender peers, 4) outcome - any relevant health and well-being outcome related to the list above, 5) setting - UK and international, with comparable policy and cultural contexts, 6) design - primary qualitative, quantitative or mixed methods studies. Studies were excluded where sexual orientation and gender identity was not clearly defined, where there were no meaningful outcomes or where papers were solely theoretical, opinions, editorials or case reports.

Searches were undertaken in four electronic databases (PubMed, Web of Science, ProQuest, and Cochrane). In addition, Google Scholar was searched accompanied by hand searching informed by existing knowledge and experience of the team, to find information that may be harder to reach, for example in grey literature reports or on government websites. Searches were limited to recent evidence because prevalence changes over time.

Search terms and appropriate synonyms (MeSH terms) for both searches (i.e. peer review and grey) included: ("LGB" OR "lesbian" OR "gay" OR "bisexual" OR “trans” OR "transgender" OR "transsexual" OR "queer" OR "non-binary" OR "MSM" OR "intersex" OR "gender identity" OR "sexual orientation" OR “gender minorit*” OR “sexual minorit*”) AND (“gambl*” OR "betting" OR "lotter*" OR "lotto*" OR "casino*" OR "loot box*").

Filters used:

- English language only
- Start date: 15.6.2000
- End date: 15.6.2023

Search string tests and the number of relevant hits for each database are reflected in an additional supplemental file. The inclusion and exclusion criteria were as follows:

| **Inclusion Criteria** | **Exclusion Criteria** |
| --- | --- |
| Written in the English language | Not written in English |
| Published between 2000 – 2023 | Published prior to 2000 |
| Sufficient focus on gambling/harms | Insufficient focus on gambling/harms |
| Sufficient focus on LGBTQ+ Communities (& MSM*) | Insufficient focus on LGBTQ+ communities (& MSM) |

^*^ *^Men who have sex with men^*

*Grey literature*

**Search 2 i**n addition to database searches of peer-review articles, detailed searches were undertaken to identify grey literature. This included government reports, third sector research, conference papers, dissertations/theses, and unpublished works. Whilst the peer-review literature searches were worldwide in scale, the grey literature synthesis was confined to the UK and other countries within the Organisation for Economic Development (OECD) to ensure a comparable legal and policy framework for interpretation. Literature was identified through i) Google Advanced Search (first 10 pages); ii) Google searches of relevant LGBTQ+/gambling charity websites; iii) reference trail searches; and iv) expert recommendations. A total of three grey literature articles were included.

Search tables for each database with the relevant search strings, filters and the number of hits per database follow:

| **TEST RESULTS (PubMed)** | **Total results** | **Number of relevant hits** |
| --- | --- | --- |
| **A) TESTS AROUND GENDER & SEXUALITY** |  |  |
| 1) ("gender minorit*" OR "sexual minorit*" OR "Lesbian" or "Gay" or "Bi*" OR "trans*" OR "queer" OR "non-binary") AND ("gambl*" OR "betting" OR "lotter*" OR "lotto*" OR "casino*") | 3837 |  |
| 2) ("gender minorit*" OR "sexual minorit*" OR "LGB" OR "lesbian" OR "gay" OR "bisexual" OR "transgender" OR "queer" OR "non-binary") AND ("gambl*" OR "betting" OR "lotter*" OR "lotto*" OR "casino*") | 4327 |  |
| 3) ("gender minorit*" OR "sexual minorit*" OR "LGB" OR "lesbian" OR "gay" OR "bisexual" OR "transgender" OR "queer" OR "non-binary" OR "MSM" OR "intersex") AND ("gambl*" OR "betting" OR "lotter*" OR "lotto*" OR "casino*") | 2879 |  |
| 4) ("gender minorit*" OR "sexual minorit*" OR "LGB" OR "lesbian" OR "gay" OR "bisexual*" OR "transgend*" OR "transsex*" OR "queer" OR "non binary" OR "non-binary" OR "asexual*" OR "pansexual*" OR "sexual orient*" OR "gender identit*" OR "sexual identit*" OR "gender dysphori* OR "gender nonconform*" OR "MSM") AND ("gambl*" OR "betting" OR "lotter*" OR "lotto*" OR "casino*") | 1170 |  |
| **B) TESTS AROUND GAMBLING** |  |  |
| 1) ("gender minorit*" OR "sexual minorit*" OR "LGB*" OR "lesbian" OR "gay" OR "bisexual" OR "transgender" OR "queer" OR "non-binary" OR "MSM" OR "intersex") AND ("gambl*" OR "betting*" OR "lotter*" OR "lotto*" OR "casino*" OR "scratch" OR "poker" OR "bingo" OR "slot machine") | 3008 |  |
| **Filters:** Full text, Title or Abstract, English, from 2000/6 - 2023/6 | **Relevant Total** | **111** |
|  |  |  |
| **C) DUAL TESTS** | **Number of hits** |  |
| 1) ("LGB*") AND ("gambl*" OR "betting*") | 2173 |  |
| 2) ("LGB*" OR "LGBT*") AND ("gambl*" OR "betting*") | 4365 |  |
| 3) ("LGB*" OR "lesbian" OR "gay" OR "bisexual" OR "transgender" OR "queer" OR "non-binary" OR "MSM" OR "intersex") AND ("gambl*" OR "betting*") | 31 |  |
| 6) ("LGB*" OR "lesbian" OR "gay" OR "bisexual" OR "transgender" OR "transsexual" OR "queer" OR "non-binary" OR "MSM" OR "intersex" OR "gender identity" OR "sexual orientation") AND ("gamble" OR "gambling" OR "betting" OR "lotter*" OR "lotto*" OR "casino*" or "loot box*") | 107 | 111 |
| **Filters** |  |  |
| Filters: Full text, Title or Abstract, English, from 2000/6 - 2023/6 | **Relevant Total** | **18** |
|  |  |  |
| **TEST RESULTS (Web of Science)** | **Total results** |  |
| 1) (gender minorit* OR sexual minorit* OR LGB* OR trans OR queer OR non-binary) AND (gambl* OR betting OR lotter* OR lotto* OR casino*) | 7,870 |  |
| 2) (gender minorit* OR sexual minorit* OR LGB* OR lesbian OR gay OR bisexual OR transgender OR queer OR non-binary) AND (gambl* OR betting OR lotter* OR lotto* OR casino*) | 8,764 |  |
| 3) (gender minorit* OR sexual minorit* OR LGB* OR lesbian OR gay OR bisexual OR transgender OR queer OR non-binary OR MSM OR intersex) AND (gambl* OR betting OR lotter* OR lotto* OR casino*) | 17,212 |  |
| 4) (gender minorit* OR sexual minorit* OR LGB* OR lesbian OR gay OR bisexual* OR transgend* OR transsex* OR queer OR non binary OR non-binary OR asexual* OR pansexual* OR sexual orient* OR gender identit* OR sexual identit* OR gender dysphori* OR gender nonconform* OR MSM) AND (gambl* OR betting OR lotter* OR lotto* OR casino*) | 21,243 |  |
| **B) TESTS AROUND GAMBLING** |  |  |
| 1) (gender minorit* OR sexual minorit* OR LGB* OR lesbian OR gay OR bisexual OR transgender OR queer OR non-binary OR MSM OR intersex) AND (gambl* OR betting* OR lotter* OR lotto* OR casino* OR scratch OR poker OR bingo OR slot machine) |  |  |
| **Filters:** Article, Full text, Open Access, Research areas, English, from 2000/6 - 2023/6 | **Relevant Total** | **106** |
| **TEST RESULTS (Cochrane)** |  |  |
| **A) TESTS AROUND GENDER & SEXUALITY** | **Total results** |  |
| 1) (gender minorit* OR sexual minorit* OR LGB* OR trans OR queer OR non-binary) AND (gambl* OR betting OR lotter* OR lotto* OR casino*) | 3795 |  |
| 2) (gender minorit* OR sexual minorit* OR LGB* OR lesbian OR gay OR bisexual OR transgender OR queer OR non-binary) AND (gambl* OR betting OR lotter* OR lotto* OR casino*) | 9615 |  |
| 3) (gender minorit* OR sexual minorit* OR LGB* OR lesbian OR gay OR bisexual OR transgender OR queer OR non-binary OR MSM OR intersex) AND (gambl* OR betting OR lotter* OR lotto* OR casino*) | 8790 |  |
| 4) (gender minorit* OR sexual minorit* OR LGB* OR lesbian OR gay OR bisexual* OR transgend* OR transsex* OR queer OR non binary OR non-binary OR asexual* OR pansexual* OR sexual orient* OR gender identit* OR sexual identit* OR gender dysphori* OR gender nonconform* OR MSM) AND (gambl* OR betting OR lotter* OR lotto* OR casino*) | 7709 |  |
| **B) TESTS AROUND GAMBLING** |  |  |
| 1) (gender minorit* OR sexual minorit* OR LGB* OR lesbian OR gay OR bisexual OR transgender OR queer OR non-binary OR MSM OR intersex) AND (gambl* OR betting* OR lotter* OR lotto* OR casino* OR scratch OR poker OR bingo OR slot machine) | 84 |  |
|  |  |  |
| **Filters:** Cochrane reviews, Trials, Special collections, Search variations, Cochrane group, between 2000/6 - 2023/6 | **Relevant Total** | **7** |

| **TEST RESULTS (Google Scholar)** | **Total results** | **No. of relevant hits (first 10 pages)** |
| --- | --- | --- |
| **A) TESTS AROUND GENDER & SEXUALITY** |  |  |
| 1) ("gender minorit*" OR "sexual minorit*" OR "LGB*" OR "trans" OR "queer" OR "non-binary") AND ("gambl*" OR "betting" OR "lotter*" OR "lotto*" OR "casino*") | 130,000 | 0 |
| 2) ("gender minorit*" OR "sexual minorit*" OR "LGB*" OR "lesbian" OR "gay" OR "bisexual" OR "transgender" OR "queer" OR "non-binary") AND ("gambl*" OR "betting" OR "lotter*" OR "lotto*" OR "casino*") | 57,900 | 1 |
| 3) ("gender minorit*" OR "sexual minorit*" OR "LGB*" OR "lesbian" OR "gay" OR "bisexual" OR "transgender" OR "queer" OR "non-binary" OR "MSM" OR "intersex") AND ("gambl*" OR "betting" OR "lotter*" OR "lotto*" OR "casino*") | 59,300 | 1 |
| 4) ("gender minorit*" OR "sexual minorit*" OR "LGB*" OR "lesbian" OR "gay" OR "bisexual*" OR "transgend*" OR "transsex*" OR "queer" OR "non binary" OR "non-binary" OR "asexual*" OR "pansexual*" OR "sexual orient*" OR "gender identit*" OR "sexual identit*" OR "gender dysphori* OR "gender nonconform*" OR "MSM") AND ("gambl*" OR "betting" OR "lotter*" OR "lotto*" OR "casino*") | 277,000 | 0 |
| **B) TESTS AROUND GAMBLING** |  |  |
| 1) ("gender minorit*" OR "sexual minorit*" OR "LGB*" OR "lesbian" OR "gay" OR "bisexual" OR "transgender" OR "queer" OR "non-binary" OR "MSM" OR "intersex") AND ("gambl*" OR "betting*" OR "lotter*" OR "lotto*" OR "casino*" OR "scratch" OR "poker" OR "bingo" OR "slot machine") | 143,000 | 0 |
| **C) DUAL TESTS** |  |  |
| 1) ("LGB*") AND ("gambl*" OR "betting*") | 182 | 4 |
| 2) ("LGB*" OR "LGBT*") AND ("gambl*" OR "betting*") | 1450 | 2 |
| 3) ("LGB*" OR "lesbian" OR "gay" OR "bisexual" OR "transgender" OR "queer" OR "non-binary" OR "MSM" OR "intersex") AND ("gambl*" OR "betting*") | 26,500 | 0 |
| 4) ("LGB*" OR "lesbian" OR "gay" OR "bisexual" OR "transgender" OR "queer" OR "non-binary" OR "MSM" OR "intersex") AND ("gamble" OR "gambling" OR "betting*") | 143,000 | 8 |
| 5) ("LGB*" OR "lesbian" OR "gay" OR "bisexual" OR "transgender" OR "transsexual" OR "queer" OR "non-binary" OR "MSM" OR "intersex" OR "gender identity" OR "sexual orientation") AND ("gamble" OR "gambling" OR "betting" OR "lotter*" OR "lotto*" OR "casino*") | 180,000 | 14 |
| 6) ("LGB*" OR "lesbian" OR "gay" OR "bisexual" OR "transgender" OR "transsexual" OR "queer" OR "non-binary" OR "MSM" OR "intersex" OR "gender identity" OR "sexual orientation") AND ("gamble" OR "gambling" OR "betting" OR "lotter*" OR "lotto*" OR "casino*" or "loot box*") | 184000 | 71 |
|  |  |  |
| **Filters** |  |  |
| Filters: Full text, Title or Abstract, English, from 2000/6 - 2023/6 | **Relevant total** | **101** |

**Data extraction, synthesis, and ethics**

Once studies were identified via the searches, database management software (EndNote) was used to allow storage of the primary research citations, to keep track of them, to identify included and excluded studies, and to detect duplicates. A master table was created in Word containing key information from each of the selected studies including health topic, time range of year published, geographical scope, the LGBTQ+ sub-population, methods employed, scientific journal or grey literature etc. Whilst there is ongoing deliberation in the literature regarding the need for quality assessment of included studies in the scoping review process (Booth 2007; Crowe & Sheppard, 2011; Carroll & Booth, 2015), critical appraisal of the literature is important to enable identification of strengths and limitations of the evidence base.

***Synthesis***

The included articles were combined to form a thematic construction based on the review questions. Themes formed an analytic framework to provide an overview of the breadth of the literature. The thematic analysis is presented as a narrative synthesis.

***Data storage and ethics***

All review data generated was stored at the School of Sport and Health Sciences, University of Brighton securely against unauthorised access using a password protected network and in compliance with data protection legislation. Only the review team had access to this data. To mitigate against the unlikely loss of data, copies of the digital files were backed up daily to university external (secured) servers. No ethical approval was required as primary research data were not collected.
