# Supplementary material for: What is known about gambling in lesbian, gay, bisexual, trans and queer (LGBTQ+) communities? A scoping review
Source: BMJ Open. 2025 Sep 14;15(9):e096792. doi: 10.1136/bmjopen-2024-096792 (PMC12434734; doi:10.1136/bmjopen-2024-096792)
Supplement: online supplemental appendix 2 [file bmjopen-15-9-s002.docx]

Appendix 2 CASP quality assessment of quantitative (peer-reviewed) studies

| **No** | **Study** | **1** | **2** | **3** | **4** | **5** | **6** | **7** | **8** | **9** | **10** |
| --- | --- | --- | --- | --- | --- | --- | --- | --- | --- | --- | --- |
| 1 | Birch (2015) | y | y | Y | y | y | n/a | n/a | y | y | y |
| 2 | Broman (2018) | y | y | Y | y | y | n/a | n/a | y | y | y |
| 3 | Broman (2022) | y | y | Y | y | y | n/a | n/a | y | y | y |
| 4 | Bush (2021) | y | y | Y | y | y | n/a | n/a | y | y | y |
| 5 | Grant (2023) | y | y | Y | y | y | n/a | n/a | y | y | y |
| 6 | Grant (2006) | y | y | Y | y | y | ct | ct | n | n | n |
| 7 | Hershberger (2005) | y | ct | ct | y | y | y | y | y | y | ct |
| 8 | Honrado (2023) | y | y | ct | y | y | n/a | n/a | y | y | ct |
| 9 | Klein (2014) | y | y | ct | y | y | n/a | n/a | y | y | y |
| 10 | Mathy (2002) | y | y | ct | y | y | n/a | n/a | y | y | y |
| 11 | Mattelin (2022) | y | y | Y | y | y | y | y | y | y | y |
| 12 | Noel (2022a) 'Correlates…' | y | y | Y | y | y | n/a | n/a | y | y | y |
| 13 | Noel (2022b) 'Gambling…' | y | y | y | y | y | n/a | n/a | y | y | y |
| 14 | Richard (2019) | y | y | y | y | y | n/a | n/a | y | y | y |
| 15 | Rider (2019) | y | y | y | n | n | n/a | n/a | y | y | y |
| 16 | Wicki (2021) | y | y | y | y | y | n/a | n/a | y | y | y |

*Checklist questions adapted (*as most of the included studies were observational studies without a specific exposure and outcome, we combined the following two CASP items "was the exposure accurately measured to minimise bias" and was the outcome accurately measured to minimise bias" to include an item was "gambling accurately measured to minimise bias”)* *from CASP quality assessment of cohort studies were: 1. Did the study address a clearly focused issue? 2. Was the cohort recruited in an acceptable way? 3. Was gambling accurately measured to minimise bias? 4. Have the authors identified all important confounding factors? 5. Have they taken account of the confounding factors in the design and/or analysis? 6. Was the follow up of subjects complete enough? 7. Was the follow up of subjects long enough? 8. Do you believe the results? 9. Can the results be applied to the local population? 10. Do the results of this study fit with other available evidence? Abbreviations: y—yes; ct—cannot tell; n—no; n/a—not applicable. A full appraisal is available from the authors on request.*
