# Supplementary material for: What is known about gambling in lesbian, gay, bisexual, trans and queer (LGBTQ+) communities? A scoping review
Source: BMJ Open. 2025 Sep 14;15(9):e096792. doi: 10.1136/bmjopen-2024-096792 (PMC12434734; doi:10.1136/bmjopen-2024-096792)
Supplement: online supplemental appendix 3 [file bmjopen-15-9-s003.docx]

Appendix 3 Summary of included peer reviewed and grey literature papers^[[1]](#footnote-1)^

| **Author** | **Date** | **Country** | **Title** | **Methods** | **Sample (n)** | **Aims** | **Peer Reviewed Literature**  **Findings** |
| --- | --- | --- | --- | --- | --- | --- | --- |
| 1. Birch et al | 2015 | Australia | Examining gambling & mental health in LGBTI communities: A preliminary study | Cross-sectional survey. Gambling Disorder Measure (GAM-DS) | n=69 (100% LGBTQ+).  Ages: 18 – 72 years | To explore ‘problematic’ levels of gambling in lesbian, gay, bisexual, transsexual and intersex (LGBTI) communities in NSW Australia | 20% of participants met the criteria for ‘problematic gambling.’ Most common types of gambling were pub slot machines/games (58%) followed by scratch cards (43.5%). |
| 2. Broman & Hakansson | 2018 | Sweden | Problematic Gaming and Internet Use but Not Gambling May Be Overrepresented in Sexual Minorities - A Pilot Population Web Survey Study | Cross-sectional survey. NODS-CLiP 'problem gambling' | n=605 (90% heterosexual; 10% sexual/gender minority)  Ages: 15+ years | To assess whether ‘problematic gambling’, gaming and internet use may be more common in individuals with a non-heterosexual orientation. | ‘Problematic gaming’ and internet use, but not ‘problematic gambling’, may be more common in non-heterosexual populations. |
| 3. Broman et al | 2022 | England Poland Switzerland, Italy Spain Denmark Sweden | Gambling, Gaming, and Internet Behavior in a Sexual Minority Perspective. A Cross-Sectional Study in Seven European Countries | Cross-sectional survey. NODS-CLiP 'problem gambling' | n=10,983 (7.1% with sexual minority status; n=774)  Ages: 15 – 60+ years | To investigate 'problem gambling', problem gaming and problematic internet behaviour in a European context and ascertain if it is affected by sexual orientation status. | No difference in gambling, gaming and internet behaviour among heterosexual and sexual minority men. Sexual minority women were associated with ‘problematic gambling’ and gaming behaviour. When also controlling for psychological distress, women defined as having another sexual minority status than lesbian and bisexual remained significant for ‘problematic gaming behaviour.’ |
| **Author** | **Date** | **Country** | **Title** | **Methods** | **Sample (n)** | **Aims** | **Peer Reviewed Literature**  **Findings** |
| 4. Bush et al | 2021 | Australia | Risk and protective factors for the development of gambling-related harms and problems among Australian sexual minority men | Cross-sectional survey. Gambling-related harms as measured by the Short Gambling Harms Screen; 'Problem gambling' as measured by the Problem Gambling Severity Index; Gambling behaviours assessment modelled after the Social and Economic Impact Study of Gambling in Tasmania and Victorian Prevalence Survey 2014; Gambling cognitions as measured by the Gambling Related Cognition Scale and Gambling Expectancy Questionnaire | n=101 sexual minority men (n=207 heterosexual men)  Mean ages provided – SMM (28.5 years) & Heterosexual men (26.4 years) | To compare gambling behaviour among sexual minority men (SMM) and examine potential risk factors (‘erroneous gambling cognitions’, ‘gambling outcome expectancies’, ‘hazardous alcohol use’, ‘impulsivity’, and ‘psychological’ distress; as well as perceived stigma and discrimination for the sexual minority participants) and potential protective factors (resilience, social support, and community connectedness) for 'problem gambling' severity and gambling-related harms among SMM living in Australia. | Sexual minority men had significantly lower levels of ‘problem gambling severity’ compared with heterosexual men, and report significantly lower gambling participation, frequencies and expenditure on any gambling activity. However, in the sexual minority group, 38.3% were classified in the ‘problem gambling’ category of the Problem Gambling Severity Index and 27.6% were classified in the ‘moderate-risk’ gambling category. |
| 5. Grant & Potenza | 2006 | U.S. | Sexual orientation of men with pathological gambling: prevalence and psychiatric comorbidity in a treatment-seeking sample | Cross-sectional study. 'Problem gambling' measured through a clinician-administered Structured Clinical Interview for Pathologic Gambling | n=105 (22 of which were gay or bisexual men)    Ages: 21 – 75 years | Examination of the sexual orientation and clinical correlates of men with ‘pathological gambling’ (PG). Gay and bisexual men with PG were compared with heterosexual men in terms of ‘gambling symptoms, impairment, and co-occurring psychiatric disorders’. | Gay and bisexual men vs heterosexual men were more likely to have a lifetime (81.8% vs 44.6%; 9.7; P =.002) or current prevalence of ‘pathological gambling’ (68.2% vs 34.9%; 7.9; P =.005) |
| **Author** | **Date** | **Country** | **Title** | **Methods** | **Sample (n)** | **Aims** | **Peer Reviewed Literature**  **Findings** |
| 6. Grant & Chamberlain | 2023 | U.S. | Does gambling differ in people with a minority sexual orientation? | Cross-sectional study. Structured Clinical Interview for Pathological Gambling adapted for DSM-5; the Yale-Brown  Obsessive-Compulsive Scale Modified for Pathological Gambling (PG-YBOCS) | n=534 (51 of participants were LGB; 9.6%)  Ages: 18 – 29 years | To compare LGB individuals with non-LGB individuals in terms of gambling and associated characteristics. | Clinical and neurocognitive evaluations with n=534 participants who gambled at least 5 times in the preceding year. LGB participants showed significantly higher levels of ‘problem gambling’. |
| 7. Hershberger & Bogaert | 2005 | U.S. | Male and female sexual orientation differences in gambling | Secondary analysis of cross-sectional survey interviews. Gambling frequency as measured by a 2-item unvalidated measure | n=10,598 (n=1,210 'homosexual’ men and women)  Ages: Mean ages provided - 'Homosexual’ men (30.2 years); Heterosexual men (29.23 years); 'Homosexual’ women (33.74 years); Heterosexual women (28.77 years) | To explore whether sexual orientation differences in gambling exist | Results showed that (a) ‘homosexual’ men gambled less than heterosexual men, the greatest difference occurring at low levels of gambling frequency, and (b) ‘homosexual’ women gambled more than heterosexual women, the greatest difference occurring at high levels of gambling frequency. |
| 8. Honrado t | 2023 | U.S. | Comparing Harmful Behaviors Among Dancers According to Sexual Orientation and Gender Identity Utilizing the RISQ | Cross-sectional survey. Risky, Impulsive & Self-destructive behavior questionnaire (RISQ) | n=66 (n=39 LGBT+)  Ages: 18+ years | To examine the harmful behaviours dancers engage in according to their self-reported sexual orientation and gender identity (SOGI) | Chi-square comparing SOGI group frequency of participation within each of the RISQ behaviours revealed statistically significant difference with regards to: gambling illegally; betting on sports, horses, or other animals; and buying expensive items that cannot be afforded at the spur of the moment. |
| **Author** | **Date** | **Country** | **Title** | **Methods** | **Sample (n)** | **Aims** | **Peer Reviewed Literature**  **Findings** |
| 9. Klein & Dudley | 2014 | U.S. | Impediments to academic performance of bisexual college students. | Cross-sectional survey.  Gambling outcomes as measured by a single-item, unvalidated measure | (n = 27,774; 66.1% female) of heterosexual (n = 21,835), bisexual (n = 792), and gay/lesbian (n = 572) adults  Mean age provided = 22.25 years | To investigate health-related impediments to academic success for bisexual college students. | On all measures, with the exception of discrimination, bisexual college students reported the strongest threats to academic success of all sexual orientations. Threats included consideration of gambling impact. |
| 10. Mathy | 2003 | U.S. | Transgender identity and suicidality in a nonclinical sample: Sexual orientation, psychiatric history, and compulsive behaviors | Cross-sectional survey. 'Problem gambling' as measured by a 2-item, unvalidated measure | n=73 Trans compare to heterosexual females (n=1,083) and males (n=1,077); and ‘homosexual’ females (n=256) and males (n=356).  Ages: 19 – 58 years | To examine the relation between sexual orientation and suicidality among 73 transgender respondents, who were compared to heterosexual females and males, and ‘homosexual’ females and males. | No transgender respondent reported that alcohol, drugs, or gambling was a primary difficulty at the time. |
| 11. Mattelin et al | 2022 | Sweden | Health and health-related behaviours in refugees and migrants who self-identify as sexual or gender minority. National population-based study in Sweden | Population survey. Risk gambling defined by using the short version of the Problem Gambling Severity Index (PGSI) | n=168,952 individuals (aged 16-84 years, males: 45·9%, sexual or gender minorities: 3·1%)  Ages: 16 – 84 years | To examine health and health-related behaviours in migrant and refugee individuals who identify as sexual or gender minority, and in comparison to their heterosexual peers. | Includes three hierarchical regression analyses adjusting for age, gender, sexual orientation, impulsivity, drug use, alcohol use, and gambling. Study found that transgender participants had high odds for ‘risk gambling’ (8.62, 1.94−38.40) |
| **Author** | **Date** | **Country** | **Title** | **Methods** | **Sample (n)** | **Aims** | **Peer Reviewed Literature**  **Findings** |
| 12. Noel et al (a) | 2022 | U.S. | Gambling: A Ubiquitous Behavior Among Rhode Island's Young Adults | Cross-sectional survey. 'Problem gambling' as measured by a 3-item unvalidated measure; Gambling behavior as measured by a 4-item, untested measure of gambling frequency | n=546 total respondents (% of LGBT participants not clear)  Ages: 18 – 25 years | To assess the prevalence of gambling and 'problem gambling' in Rhode Island young adults and to identify socio-demographic correlates of gambling. | Transgender respondents had significantly higher odds of ‘gambling problem symptoms’ [95% CI] = 3.61 [1.32, 9.86]. |
| 13. Noel et al (b) | 2022 | U.S. | Correlates of gambling & gambling problems among Rhode Island young adults: A cross-sectional study | Cross-sectional survey.  'Problem gambling' as measured by a 3-item unvalidated measure; Gambling behavior as measured by a 4-item, untested measure of gambling frequency | n=540 (LGB = n=141; Trans = n=21)  Ages: 18 – 25 years | To assess four types of gambling activities - sports betting; betting on races; gaming tables at a casino; and poker machines at a casino | 11.5% of participants had ‘gambling problems’. Odds of gambling activities were higher among men; Black, Indigenous, People of Colour; older young adults; and essential workers. Odds of ‘gambling problems’ were 2.4 times higher among participants who engaged with sports betting. |
| 14. Richard et al | 2019 | Canada | Variations in Gambling Disorder Symptomatology Across Sexual Identity Among College Student-Athletes | Cross-sectional survey.  'Problem gambling' as measured using DSM-5 criteria to assess symptomology; Gambling behaviors as measured by the Gambling Activities Questionnaire | n=19,299 (LGBTQ+ = 4.25%)  College students (ages not provided) | To explore ‘gambling disorder symptomatology’ by sexual identity status – comparing differences in the severity of ‘gambling disorder symptomatology’ between sexual minority and heterosexual student-athletes. | Gay and bisexual men had ‘disordered gambling’ scores 3.42 times higher than heterosexual men (p < .01), when adjusting for race/ethnicity, and years in college. Gay/lesbian and bisexual women reported ‘disordered gambling’ scores 2.57 higher than heterosexual women (p < .01) when adjusting for race/ethnicity and years in college. |
| **Author** | **Date** | **Country** | **Title** | **Methods** | **Sample (n)** | **Aims** | **Peer Reviewed Literature**  **Findings** |
| 15. Rider et al | 2019 | U.S. | Gambling Behaviors and 'Problem Gambling': A Population-Based Comparison of Transgender/Gender Diverse and Cisgender Adolescents | Cross-sectional survey.  Gambling behavior as measured by the 3-item Brief Adolescent Gambling Screen and a 4-item, unvalidated measure | n=80,929 (n = 2168; 2.7% trans/gender diverse)  Ages: 14 – 17 years | To examine and compare gambling behaviours between transgender and gender diverse (TGD) youth and their cisgender peers. | TGD youth reported greater involvement in most gambling behaviours and ‘problem gambling’ compared to cisgender youth. In comparisons by birth-assigned sex, TGD youth assigned male at birth were particularly at risk for gambling involvement and 'problem gambling'. TGD youth assigned female at birth also reported higher rates of ‘problem gambling’ than both cisgender youth assigned male and female at birth. |
| 16. Wicki et al | 2021 | Switzer-land | Curvilinear associations between sexual orientation and problematic substance use, behavioural addictions and mental health among young Swiss men | Cross-sectional survey.  Gambling disorder as measured by DSM-5 criteria | n=5294 (LGB = 4.2%; n=217)  Mean age provided: 25.5 years | To explore ‘behavioural addictions’ among people with a minority sexual orientation. | Although there were differences across criterion variables, in general, the ‘highest risks of problematic substance use, behavioural addictions and mental health problems’ were estimated for mostly-heterosexual, bisexual or ‘mostly-homosexual’ men, followed by ‘homosexual’ men, and with heterosexual men facing the lowest risk. |

| **Author** | **Date** | **Country** | **Title** | **Method** | **Sample (n)** | **Aims** | **Grey Literature**  **Findings** |
| --- | --- | --- | --- | --- | --- | --- | --- |
| 17.  Bush et al | 2020 | Australia | Examining risk and protective factors for the development of gambling-related harms and problems in Victorian LGBTIQ+ communities | Survey & Interviews. Use of the Problem Gambling Severity Index (PGSI), the Short Gambling Harms Screen (SGHS), the Gambling Related Cognition Scale (GRCS), and the Gambling Expectancy Questionnaire (GEQ) | Survey: n=385 (n=213 cishet; n=172 LGBTIQ+)  Interviews: n=11 LGBTIQ+  Ages: 18+ years | To examine gambling in the LGBTIQ+ population, psychological factors and minority stress | LGBTIQ+ participants showed lower levels of 'problem gambling' compared to cishet participants, fewer gambling related harms, fewer friends who gambled, lower levels of ‘hazardous drinking’, higher levels of psychological distress, higher levels of impulsivity and lower levels of social support. |
| 18.  Bush-Evans | 2023 | UK | Reducing gambling harms in LGBTQ+ communities | Survey & interviews. Gambling measures not reported via policy briefing | Survey: n=321 LGBTQ+ adults  Interviews: n=20 LGBTQ+ adults  Ages: N/A | To explore the influences on gambling and gambling harms within UK-based LGBTQ+ communities. | Over two thirds (67.3%) of those who gamble experienced some level of harm (PGSI.1+), with 14.3% indicative of 'problem gambling'. Here 71% of LGBTQ+ people reported experiences of discrimination or harassment in their life, with 89% reporting experiences of isolation. The paper argues that links can be made with gambling used as a means of coping with adverse life events. |
| 19. Rotermann & Gilmour | 2022 | Canada | Who gambles and who experiences gambling problems in Canada | Cross-sectional (population) survey.  Utilised the Canadian Problem Gambling Index | n=24,983,000  Ages: 15+ years | A health population study of gambling and ‘gambling problems’ in those aged 15 and older | A multivariable analysis of males found higher odds that sexual minority men would have ‘gambling problems’ as compared to heterosexual men via an adjusted odds ratio (3.0 vs 1.0; 95% CI). However, the study found no bivariate differences in the rates of moderate-to-severe ‘gambling problems’ by sexual orientation, and no differences in rates of past-year gambling. |

1. Please note that quotation marks are used in reference to any words or phrases mentioned in the studies which are now considered to be problematic or out-of-date. [↑](#footnote-ref-1)
