# Supplementary material for: What is known about gambling in lesbian, gay, bisexual, trans and queer (LGBTQ+) communities? A scoping review
Source: BMJ Open. 2025 Sep 14;15(9):e096792. doi: 10.1136/bmjopen-2024-096792 (PMC12434734; doi:10.1136/bmjopen-2024-096792)
Supplement: Supplementary file 5 [file bmjopen-2024-096792.reviewer_comments.pdf]

PEER REVIEW HISTORY

BMJ Open publishes all reviews undertaken for accepted manuscripts. Reviewers are asked to complete a checklist review form and are provided with free text boxes to elaborate on their assessment. These free text comments are reproduced below.

ARTICLE DETAILS

Title (Provisional)

What is known about gambling in lesbian, gay, bisexual, trans and queer (LGBTQ+) communities? A scoping review

Authors

Zeeman, Laetitia; Sawyer, Alexandra; Bailey, Louis; Sherriff, Nigel; Smith, Matt

---

VERSION 1 - REVIEW

Reviewer 1

Name Donnachie, Craig

Affiliation University of Strathclyde, School of Psychological Sciences and Health

Date 28-Mar-2025

COI None

This paper reports on a scoping review conducted to summarise the available literature on gambling behaviour among LGBTQ+ populations. The scoping review incorporates a range of quantitative, qualitative and mixed methods evidence from available peer-reviewed research and wider grey literature. This is an important paper which highlights the need for further large scale 'high quality' research (both quantitative (e.g. population-based) and qualitative research (incorporating lived experience)) to establish salient risk and protective factors to gambling experienced among LGBTQ+ people that are vital to informing the development of future gambling harm prevention work and targeted interventions. I have outlined my comments and suggestions below for further consideration below and hope these are useful to the authors in developing their manuscript.

Comments

Page 3, lines 40-43: It would be useful to highlight the gambling-related harms frameworks including explication of the specific dimensions (e.g. Langham et al (2016) and Wardle et al (2018) frameworks) affecting resources, health/mental health and social relationships.

Page 3, lines 53-54: Please include in full when first introducing full pertaining to the target population - i.e. lesbian, gay, bisexual, trans, queer+ (LGBTQ+).

Page 4, lines 16-17: ‘Several gambling reviews related to sexual orientation and gender identity exist’ – Please include some examples in brackets at the end of this sentence (e.g. several references) and please provide more details on the types of reviews (e.g. systematic, scoping etc).

Page 4, lines 17-20: Further, can you include more details to critically appraise these review studies including the types of primary studies incorporated (cross sectional, qualitative etc) and the types of outcomes employed? This is important context in setting out the novel contribution and rationale for the current review study.

Page 5, lines 40-43: It would be useful for the reader to outline what the broad themes and sub-themes were explicitly here that are then presented in the narrative synthesis.

Page 13, lines 30-35: Useful to highlight and reflect explicitly on how intersectional stigma may operate given the findings presented from the various sources in relation to gambling-related harms.

Results: Generally, some of the qualitative evidence is integrated throughout (e.g. interviews) and suggest keeping all the mixed-methods and specific qualitative research evidence distinct.

**Reviewer**	**2**
**Name**	**Marionneau, Virve**
**Affiliation**	**University of Helsinki**
**Date**	**25-Apr-2025**
**COI**	**None**

This paper reviews existing research on gambling and gambling harms amongst LGBTQ+ individuals. The topic is timely and interesting. However, I am unsure what this paper adds to existing research. Two prior reviews have recently been published on the same topic, with similar results and outcomes. ("need for further research") (see refs below).

The presentation of the paper is good overall, and the review has been conducted appropriately. However, to make this paper more original would be to expand the scope of the literature search (for example, by extending the search to other countries less comparable to GB and to other languages besides English to capture a wider sample of grey literature from outside the anglosphere. Only three grey literature sources were identified in the current review).

It is unclear whether this paper has a GB focus or whether it has a global focus. The introduction focuses strongly on GB, and the review has been conducted in a way to exclude contexts that are not clearly comparable with GB. However, at the same time, one of the conclusions of the review is that there is a need for a broader contextual understanding

outside of the western world. This is contradictory. If this paper has a focus on producing information for the GB context specifically, this should be highlighted clearly in the introduction.

The use of LGBTQ+ terminology needs to be clarified. The used terms differ at different parts of the manuscript (including LGB; LGB+; LGBTQI+). It would be useful to have an overview of these different concepts in the introduction.

One of the key outcomes of the paper is that there is a need for culturally sensitive intervention and prevention programmes to address gambling harms amongst LGBTQ+ individuals. This is probably a good conclusion. However, I am wondering what these kinds of programmes would look like? The authors might want to expand this idea a bit further and make some concrete suggestions. Likewise, is there any evidence that the gambling industry is targeting LGBTQ+ individuals specifically e.g. with key symbolics?

Minor comments:

The global prevalence figures for PG in the introduction could reference a more recent systematic review by Tran et al. (2024) (Tran, L. T., Wardle, H., Colledge-Frisby, S., Taylor, S., Lynch, M., Rehm, J., ... & Degenhardt, L. (2024). The prevalence of gambling and problematic gambling: a systematic review and meta-analysis. The Lancet Public Health.)

There is conceptual unclarity with regards to gambling harm. Population studies using standardised measures such as PGSI measure problem gambling (PG) not gambling harm. When you discuss global prevalence, for example, this should be global prevalence of PG, not of gambling harm.

GB and UK are sometimes used interchangeably.

Prior reviews:

Lee, B. N., & Grubbs, J. B. (2023). Problem gambling within sexual and gender minorities: A systematic review. Addictive Behaviors, 144, 107742.

Malkin, M. L., & Stacey, M. (2024). Gambling behavior among LGBTQ+ individuals: The role of gender and gender identity. Journal of Gambling Studies, 40(2), 1037-1060.

VERSION 1 - AUTHOR RESPONSE

Reviewer: 1
Dr. Craig Donnachie, University of Strathclyde
Comments to the Author:

This paper reports on a scoping review conducted to summarise the available literature on gambling behaviour among LGBTQ+ populations. The scoping review incorporates a range of quantitative, qualitative and mixed methods evidence from available peer-reviewed research and wider grey literature. This is an important paper which highlights the need for further large

scale ‘high quality’ research (both quantitative (e.g. population-based) and qualitative research (incorporating lived experience)) to establish salient risk and protective factors to gambling experienced among LGBTQ+ people that are vital to informing the development of future gambling harm prevention work and targeted interventions. I have outlined my comments and suggestions below for further consideration below and hope these are useful to the authors in developing their manuscript.

Response:

Our thanks for taking the time to review and comment on our paper. We have responded to these suggestions below.

Reviewer: 1

Page 3, lines 40-43: It would be useful to highlight the gambling-related harms frameworks including explication of the specific dimensions (e.g. Langham et al (2016) and Wardle et al (2018) frameworks) affecting resources, health/mental health and social relationships.

Response:

Done. Thank you for these useful suggestions. We have included both the Langham et al (2016) and Wardle et al (2018) frameworks in the text.

Reviewer: 1

Page 3, lines 53-54: Please include in full when first introducing full pertaining to the target population - i.e. lesbian, gay, bisexual, trans, queer+ (LGBTQ+).

Response:

Done. As suggested, we have used these terms in full both when they are first introduced in the abstract and in the main text.

Reviewer: 1

Page 4, lines 16-17: ‘Several gambling reviews related to sexual orientation and gender identity exist’ – Please include some examples in brackets at the end of this sentence (e.g. several references) and please provide more details on the types of reviews (e.g. systematic, scoping etc).

Response:

Done. Thank you for this suggestion. Existing reviews in the field were acknowledged by including a more detailed representation of the systematic review by Lee & Grubbs 2023; and the scoping review by Devault-Tousignant et al 2023.

Reviewer: 1

Page 4, lines 17-20: Further, can you include more details to critically appraise these review studies including the types of primary studies incorporated (cross sectional, qualitative etc) and the types of outcomes employed? This is important context in setting out the novel contribution and rationale for the current review study.

Response:

Done. As suggested, further information was included to provide a succinct critical appraisal of the methods of studies included in the systematic review (Lee & Grubbs 2023) and the scoping review (Devault-Tousignant et al 2023). Further details were provided of the conflicting results on gambling in LGBTQ+ populations.

Reviewer: 1

Page 5, lines 40-43: It would be useful for the reader to outline what the broad themes and sub-themes were explicitly here that are then presented in the narrative synthesis.

Response:

Done. Thank you for this suggestion. Key themes and subthemes from across the identified studies were included as a separate paragraph to give an indication of the logical structure that will follow, prior to the Heading: *The prevalence of gambling amongst LGBTQ+ communities.*

Reviewer: 1
Page 13, lines 30-35: Useful to highlight and reflect explicitly on how intersectional stigma may operate given the findings presented from the various sources in relation to gambling-related harms.
Response:
Done. A sentence was added to the discussion section to bring awareness of intersectional stigma to the fore.

Reviewer: 1
Results: Generally, some of the qualitative evidence is integrated throughout (e.g. interviews) and suggest keeping all the mixed-methods and specific qualitative research evidence distinct.
Response:
Done. Qualitative data has now been limited to one section only entitled: "LGBTQ+ gambling lived experience" All quotations for interview data are cited in this section to ensure mixed methods results and purely qualitative data remain apart.

Reviewer: 2
Dr. Virve Marionneau, University of Helsinki
Comments to the Author:

This paper reviews existing research on gambling and gambling harms amongst LGBTQ+ individuals.
The topic is timely and interesting.
Response:
Our thanks for taking the time to review and comment on our paper. We have responded to these suggestions below.

Reviewer: 2
However, I am unsure what this paper adds to existing research. Two prior reviews have recently been published on the same topic, with similar results and outcomes. ("need for further research") (see refs below).
The presentation of the paper is good overall, and the review has been conducted appropriately.
Response:
Thank you for this comment. Since these reviews have been published (searches conducted), there has been an increase in papers exploring gambling harms in LGBTQ+ communities. For example, our review found that five papers were published in 2022. Our review includes 19 papers compared to 6 papers in the scoping review by Devault-Tousignant. Furthermore, the inclusion of grey literature identified the only qualitative research on gambling harms in LGBTQ+ communities, thus providing in-depth insight into lived experience of gambling harms, which was not explored in previous reviews therefore we are confident our review makes an important contribution to the literature.

Reviewer: 2
However, to make this paper more original would be to expand the scope of the literature search (for example, by extending the search to other countries less comparable to GB and to other languages besides English to capture a wider sample of grey literature from outside the anglosphere. Only three grey literature sources were identified in the current review).

Response:
Thank you for this suggestion. We did indeed consider this notion of expanding the scope for this review. However, our remit was limited within the funded project constraints both in terms of scope and timeframe. Moreover, the language proficiency of the review team members (predominantly English) meant it was not possible to consider papers in other languages (which is not unusual) as we did not have the budget to allow us to do this (e.g. translation costs, or engagement of additional co-investigators in other countries)

Reviewer: 2
It is unclear whether this paper has a GB focus or whether it has a global focus. The introduction focuses strongly on GB, and the review has been conducted in a way to exclude contexts that are not clearly comparable with GB.
Response:
That is a good observation thank you. The review was conceptualised based on early findings reported in GB survey data for England, Scotland and Wales. The review is therefore framed within a UK context. Due to the limited research available in the UK, we designed the scoping review to draw on broader literature beyond the UK, weighted towards research conducted in the USA., Australia, Canada and Western Europe. We have clarified in the text that gambling research in comparative legislative, policy, and cultural contexts were considered, where there was a lack of evidence in the UK. This was added to the limitations of the review.

Reviewer: 2
However, at the same time, one of the conclusions of the review is that there is a need for a broader contextual understanding outside of the western world. This is contradictory. If this paper has a focus on producing information for the GB context specifically, this should be highlighted clearly in the introduction.
Response:
Done. Thank you, we have amended the conclusion to state that more robust gambling research is needed for LGBTQ+ communities in the UK, and arguably more broadly. We have added a line to the introduction to make clear from the onset, the framing within a UK context.

Reviewer: 2
The use of LGBTQ+ terminology needs to be clarified. The used terms differ at different parts of the manuscript (including LGB; LGB+; LGBTQI+). It would be useful to have an overview of these different concepts in the introduction.
Response:
A useful observation thank you. We have used the terms such as lesbian, gay, bisexual, trans, queer and intersex+ (LGBTQI+) in full where possible, and more specifically where these terms appear for the first time. As individual studies report on specific populations insofar as LGB or LGBT+ or LGBTQI+, we have used these terms as a precise representation of findings for specific populations.

Reviewer: 2
One of the key outcomes of the paper is that there is a need for culturally sensitive intervention and prevention programmes to address gambling harms amongst LGBTQ+ individuals. This is probably a good conclusion. However, I am wondering what these kinds of programmes would look like? The authors might want to expand this idea a bit further and make some concrete suggestions.
Response:
Done. This is a useful suggestion thank you. Limited research is available on interventions to address gambling in LGBTQ+ communities. As such culturally sensitive intervention

programmes need to be developed. We have added a paragraph in the discussion section to outline broad principles of culturally sensitive programmes with key considerations for their design.

Reviewer: 2
Likewise, is there any evidence that the gambling industry is targeting LGBTQ+ individuals specifically e.g. with key symbolics?
Response:
Thank you for raising this important point. We did not come across any evidence that the gambling industry is targeting LGBTQ+ individual specifically with key symbolics, however we are aware that the UK is out of step with the rest of Europe, where an outright ban on gambling advertising is in place. The UK currently has, at the time of writing the paper, no restrictions on any gambling advertising.

Minor comments:
Reviewer: 2

The global prevalence figures for PG in the introduction could reference a more recent systematic review by Tran et al. (2024) (Tran, L. T., Wardle, H., Colledge-Frisby, S., Taylor, S., Lynch, M., Rehm, J., ... & Degenhardt, L. (2024). The prevalence of gambling and problematic gambling: a systematic review and meta-analysis. The Lancet Public Health.)
Response:
Done. Thank you for this valuable suggestion to help ensure the paper reflects the broader global context related to gambling. We have cited prevalence data from the Trans et al (2024) review in the introduction section for prevalence of gambling in the general population.

Reviewer: 2
There is conceptual unclarity with regards to gambling harm. Population studies using standardised measures such as PGSI measure problem gambling (PG) not gambling harm. When you discuss global prevalence, for example, this should be global prevalence of PG, not of gambling harm.
Response:
Done Thank you, we have undertaken a comprehensive edit to ensure accuracy in reporting evidence related to the Problem Gambling Severity Index (PGSI). We have removed reference to gambling harm in these sections.

Reviewer: 2
GB and UK are sometimes used interchangeably.
Response:
We have removed the term GB where possible and have used UK in the text. However, where we report on specific surveys conducted in GB (England, Scotland and Wales without Northern Ireland), we refer to GB instead of the UK, to accurately reflect the survey population. We have clarified this in the text.

As for the papers below, thank you for these suggestions. Where they were not already included in the text, we have incorporated these publications in both the text and the reference list.

Prior reviews:

Lee, B. N., & Grubbs, J. B. (2023). Problem gambling within sexual and gender minorities: A systematic review. Addictive Behaviors, 144, 107742.

Malkin, M. L., & Stacey, M. (2024). Gambling behavior among LGBTQ+ individuals: The role of gender and gender identity. Journal of Gambling Studies, 40(2), 1037-1060.

Reviewer: 1
If you have selected 'Yes' above, please provide details of any competing interests.: Not applicable.

Reviewer: 2
If you have selected 'Yes' above, please provide details of any competing interests.: Not applicable

---

VERSION 2 - REVIEW

Reviewer 1

Name Donnachie, Craig

Affiliation University of Strathclyde, School of Psychological Sciences and Health

Date 03-Jul-2025

COI

I have no further comments to the author other than that I enjoyed reading the revised paper and satisfied they have addressed all of my initial comments and concerns.

Reviewer 2

Name Marionneau, Virve

Affiliation University of Helsinki

Date 22-Jun-2025

COI

Thank you to the authors for a thorough revision! The paper has improved significantly.

I only have some relatively minor comments.

Introduction:

- What is the reference for the sentence on the growth of gambling globally (third sentence of the introduction)

- Is the sentence 'The prevalence of 'problem gambling'gambling harm varies between international settings with prevalence estimates for the general adult population raging

between 0.12% to 5.8% during 2000 – 2015.(2)' needed anymore since you have added the more up-to-date reference (Tran et al)?

- The Tran et al., Devault-Toisignant et al. and Lee & Grubbs references are not numbered but appear in the text in a written form.

- The last paragraph of the introduction is very long – you might consider splitting it.

Methods:

- As you well describe in your responses, there has been a significant increase in the volume of literature on gambling in the LGBT+ communities. The current review covers literature until June 2023. As this is already two years ago, could it be possible for you to consider updating the search? Updated systematic searches are quite common in these types of studies and they are not usually too time-consuming to perform.

Results:

- There is a new paragraph just before the prevalence section that outlines the five themes of the analysis. These are not the same four as under the 'aims of the review' section in the introduction but do cover the same issues. Would it be possible to streamline these two? Or could the authors explain if there is a theme that you found that was not identified in the aims?

Discussion

- Reference to Zeeman et al. is written rather than numbered

VERSION 2 - AUTHOR RESPONSE

Reviewer: 1
Dr. Craig Donnachie, University of Strathclyde
Comments to the Author:
I have no further comments to the author other than that I enjoyed reading the revised paper and satisfied they have addressed all of my initial comments and concerns.

Response:
Thank you.

Reviewer: 2
Dr. Virve Marionneau, University of Helsinki
Comments to the Author:
Thank you to the authors for a thorough revision! The paper has improved significantly.

I only have some relatively minor comments.

Introduction:
- What is the reference for the sentence on the growth of gambling globally (third sentence of the introduction)

Response:
We have updated Reference [2] to cite the most recent Polaris Market Research report as follows:
Polaris Market Research. Online gambling market share, size, share, trends, industry analysis report: By type (sports betting, casinos, poker, bingo, and, others), by device and by region - Market; segment forecast, 2025-2034. Polaris Market Research; 2025 [Available from: https://www.polarismarketresearch.com].

- Is the sentence 'The prevalence of 'problem gambling' gambling harm varies between international settings with prevalence estimates for the general adult population raging between 0.12% to 5.8% during 2000 – 2015.(2)' needed anymore since you have added the more up-to-date reference (Tran et al)?

Response:
Done. The prevalence estimates for 2000-2015 were removed as suggested.

- The Tran et al., Devault-Toisignant et al. and Lee & Grubbs references are not numbered but appear in the text in a written form.
Response:
Done. These studies for Devault-Toisignant et al. and Lee & Grubbs are now numbered in the text as per the suggestion.

- The last paragraph of the introduction is very long – you might consider splitting it.

Response:
Thank you for this suggestion. The last paragraph was shortened to improve clarity.

Methods:
- As you well describe in your responses, there has been a significant increase in the volume of literature on gambling in the LGBT+ communities. The current review covers literature until June 2023. As this is already two years ago, could it be possible for you to consider updating the search? Updated systematic searches are quite common in these types of studies and they are not usually too time-consuming to perform.

Response:
This is a constructive suggestion, and we would have liked to update the scoping review, however the review was undertaken in 2023 as part of a commissioned study. The scoping review was submitted to BMJ Open in Nov 2024, and we have specified the timeframe for searches up to 2023. As much as we would like to, we do not have the capacity to re-write the content and the references list with any additional papers published in the last two years.

Results:
- There is a new paragraph just before the prevalence section that outlines the five themes of the analysis. These are not the same four as under the 'aims of the review' section in the introduction but do cover the same issues. Would it be possible to streamline these two? Or could the authors explain if there is a theme that you found that was not identified in the aims?

Response:
Done. The aims of the review and the identified themes were edited to ensure they are more closely aligned.

Discussion
- Reference to Zeeman et al. is written rather than numbered

Response:
Done. We have ensured that all references are numbered in the text with the accompanying references following in full via the reference list.

Reviewer: 1
If you have selected 'Yes' above, please provide details of any competing interests.: Not applicable.

Reviewer: 2
If you have selected 'Yes' above, please provide details of any competing interests.: Not applicable

---

VERSION 3 - REVIEW

**Reviewer**	**2**
**Name**	**Marionneau, Virve**
**Affiliation**	**University of Helsinki**
**Date**	**15-Aug-2025**
**COI**	

I have no further comments, I think the authors have done a really good job addressing all my comments.
